# Low protein diet protects the liver from Salmonella Typhimurium-mediated injury by modulating the mTOR/autophagy axis in macrophages

**DOI:** 10.1038/s42003-024-06932-w

**Published:** 2024-09-30

**Authors:** Edyta E. Wojtowicz, Katherine Hampton, Mar Moreno-Gonzalez, Charlotte L. Utting, Yuxuan Lan, Paula Ruiz, Gemma Beasy, Caitlin Bone, Charlotte Hellmich, Rebecca Maynard, Luke Acton, Matthew Markham, Linda Troeberg, Andrea Telatin, Robert A. Kingsley, Iain C. Macaulay, Stuart A. Rushworth, Naiara Beraza

**Affiliations:** 1 Earlham Institute, Cellular Genomics Strategic Programme, Norwich Research Park, Norwich, UK, Norwich Research Park, Norwich, UK; 2 Metabolic Health Research Centre, Faculty of Medicine, University of East Anglia, Norwich Research Park, Norwich, UK; 3 Gut Microbes and Health Institute Strategic Programme, Quadram Institute Bioscience, Norwich Research Park, Norwich, UK; 4 Food, Microbiome and Health Institute Strategic Programme, Quadram Institute Bioscience, Norwich Research Park, Norwich, UK; 5 Microbes and Food Safety Institute Strategic Programme, Quadram Institute Bioscience, Norwich Research Park, Norwich, UK; 6 Department of Haematology, Norfolk and Norwich University Hospital, Norwich, UK; 7 Science Operations, Quadram Institute Bioscience, Norwich Research Park, Norwich, UK; 8 School of Biological Sciences, University of East Anglia, Norwich Research Park, Norwich, UK

**Keywords:** Monocytes and macrophages, Infection

## Abstract

Western diets are the underlying cause of metabolic and liver diseases. Recent trend to limit the consumption of protein-rich animal products has become more prominent. This dietary change entails decreased protein consumption; however, it is still unknown how this affects innate immunity. Here, we studied the influence of a low protein diet (LPD) on the liver response to bacterial infection in mice. We found that LPD protects from *Salmonella enterica* serovar Typhimurium *(S*. Typhimurium*)*-induced liver damage. Bulk and single-cell RNA sequencing of murine liver cells showed reduced inflammation and upregulation of autophagy-related genes in myeloid cells in mice fed with LPD after *S*. Typhimurium infection. Mechanistically, we found reduced activation of the mammalian target of rapamycin (mTOR) pathway, whilst increased phagocytosis and activation of autophagy in LPD-programmed macrophages. We confirmed these observations in phagocytosis and mTOR activation in metabolically programmed human peripheral blood monocyte-derived macrophages. Together, our results support the causal role of dietary components on the fitness of the immune system.

## Abstract (web summary)

Low protein diet protects against *Salmonella* Typhimurium-mediated liver injury in mice corresponding to reduced mTOR activation and increased autophagy in macrophages. mTOR pathway activation with amino acid supplementation reverses this protection.

## Introduction

Metabolism and immunity share a close connection at both the cellular and organismal levels. Metabolic regulation of the innate and adaptive immune responses is an active and expanding area of investigation. In particular, the roles of dietary choices in regulating the functions of different immune cells^1^, and as the underlying cause of chronic diseases^2^ are increasingly recognised.

Western diets rich in processed foods are linked to metabolic diseases. They also cause altered composition and functional states of various immune cells in tissues, contributing to chronic activation of macrophages and inflammation^3–5^. Epidemiological studies have revealed an increased susceptibility to infections in patients with diabetes or obesity, pointing to an evident dysfunction in the immune response^6,7^.

On the opposite side of the Western-diet spectrum, a part of the population is adopting healthier dietary habits that include reduced consumption of meat products and increased consumption of plant-based products. This nutritional adjustment encompasses a significantly reduced intake of protein and amino acids^8^. Protein malnutrition (0.5-2.5% protein) has adverse consequences in young children and juvenile mice for the immune system function^9–12^, leading to immunosuppression^11,12^, while a reduction in dietary protein (7–10% protein) inhibits cancer development^13,14^ and metastasis^15^. Still, our knowledge on whether the physiological reduction of protein intake impacts host immunity and response to infection is limited.

To rapidly identify pathogens, cells of the innate immune system, i.e. macrophages, phagocytose bacteria and destroy them in phagosomes^16^. Additionally, macrophages secrete proinflammatory and antimicrobial mediators to inactivate pathogens^17,18^. Bacteria, including *Salmonella enterica* serotype Typhimurium (*S*. Typhimurium), have developed mechanisms to escape innate immunity through macrophage necroptosis^19,20^ or circumventing autophagy, by directing for degradation molecular sensors of mitochondrial stress and energy level-AMP activated protein kinase (AMPK) and sirtuin1 (SIRT1)^21^. Both proteins regulate the evolutionary conserved nutrient sensing pathway-mammalian target of rapamycin (mTOR)^22^. mTOR integrates the metabolic, autophagic and phagocytic state of the cell, therefore linking innate immunity with the host metabolic state^23^.

Dietary nutrients, more specifically amino acids (in particular serine, glutamine, leucine and arginine), regulate innate immunity, specifically they shift the balance between proinflammatory (M1-like) or pro-healing (M2-like)^24^ macrophage subsets. Depletion of glutamine and serine promotes expression of IL-1b^25–27^ and increased abundance of M1 macrophages, while the inhibition of serine synthesis decreases IL-1b and TNF production in LPS-induced endotoxemia^28^. Arginine depletion induces M2-like macrophages, facilitating proliferation and healing^29^. Leucine abundance activates the metabolic master regulator mTOR (specifically the mTORC1 subunit) by providing the acetyl group^30^, enhancing glycolysis and proinflammatory M1 macrophage state. mTOR activation also leads to the inflammasome activation that mediate the release of proinflammatory cytokines^31^, while shutting down autophagy, thus promoting survival of pathogens like *S*. Typhimurium within macrophages^32^.

Despite this body of evidence on the key role of amino acids for fine tuning immune response, it is unknown how a systemic lower abundance of dietary amino acids regulates host innate immune response to pathogen infection.

In the present study, we test the hypothesis that a LPD affects the host liver response to *S*. Typhimurium infection by the transcriptional and functional reprogramming of macrophages in the liver.

Our results show that a LPD diet promotes an expansion and metabolic reprogramming of myeloid cells in the liver, rendering them anti-inflammatory while more efficient phagocytosis and autophagy in response to *S*. Typhimurium infection. Here, we provide mechanistic insights into the beneficial role of a LPD on bacteria clearance and preserving liver function after infection.

## Results

### Low protein diet protects liver function from *S*. Typhimurium induced damage

**Fig. 1:**
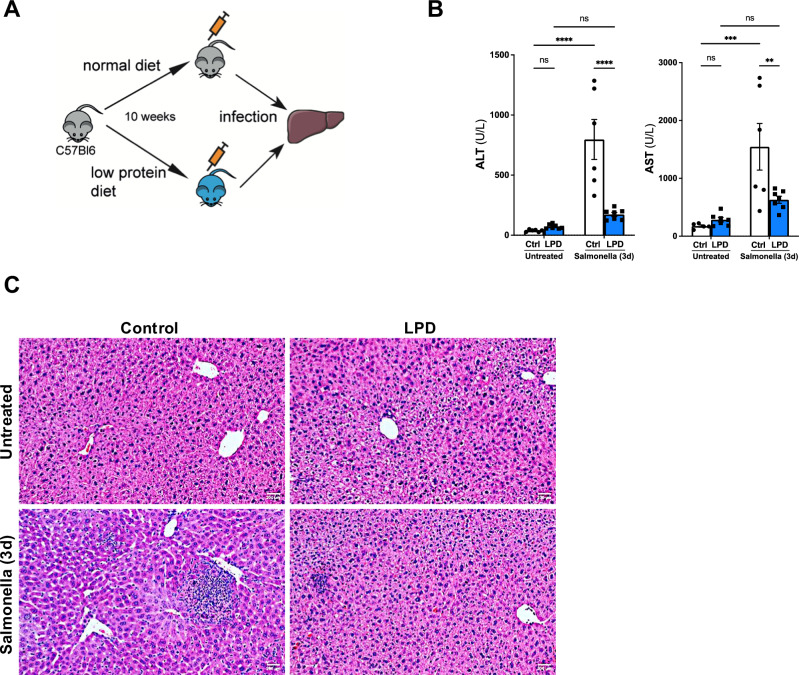
Low protein diet feeding limits *S*. Typhimurium induced liver damage in vivo. **A** Experimental set up scheme. **B** Quantification of the serum levels of alanine aminotransferase (ALT) (Control diet after Salmonella vs LPD after Salmonella *p* < 0.0001; Ctrl untreated vs Ctrl Salmonella *p* < 0.0001; applied two-way ANOVAtest) and aspartate aminotransferase (AST) (Control diet after Salmonella vs LPD after Salmonella *p* = 0.0031; Ctrl untreated vs Ctrl Salmonella *p* < 0.0002; applied two-way ANOVA test) in animals fed with a normal or low protein diet (LPD) for 10 weeks and 3 days after *S*. Typhimurium infection. **C** H&E staining in liver sections obtained from control and LPD fed mice infected with *S*. Typhimurium. Arrows point to necrotic areas. Analyses were done from *n* = 5–8 mice. Results shown are representative from 3 independent experiments. Representative microscopic images are shown from 20x magnification. Values are mean ± SEM.

To evaluate the impact of reduced protein intake on the liver response to infection we fed C57BL/6J mice *ad libitum*, a normal (control, 23% protein content) or a low protein (LPD, 6% protein content) isocaloric diets for 10 weeks after which, a group of mice per feeding regime were inoculated with *S*. Typhimurium (Fig. 1A). We confirmed that LPD feeding had no significant effects on the circulating levels of alanine aminotransferase (ALT) and aspartate aminotransferase (AST) (Fig. 1B). As expected, at three days after the inoculation of *S*. Typhimurium, we observed a significant increase in the serum levels of ALT and AST in mice fed with a control diet, while mice receiving a LPD showed significantly lower ALT and AST levels after infection, suggesting decreased liver injury compared to control diet fed animals (Fig. 1B). Histopathological analysis of liver sections with hematoxylin and eosin (H&E) staining confirmed the absence of apparent histological changes in the livers from mice receiving LPD compared to control diet (Fig. 1C, upper panels). Importantly, H&E staining revealed reduced areas of necrosis consistent with attenuated liver injury in LPD fed mice compared to normal diet after *S*. Typhimurium infection (Fig. 1C, lower panels).

### LPD attenuates expression of proinflammatory cytokines and chemokines in liver cells after *S*. Typhimurium infection

**Fig. 2:**
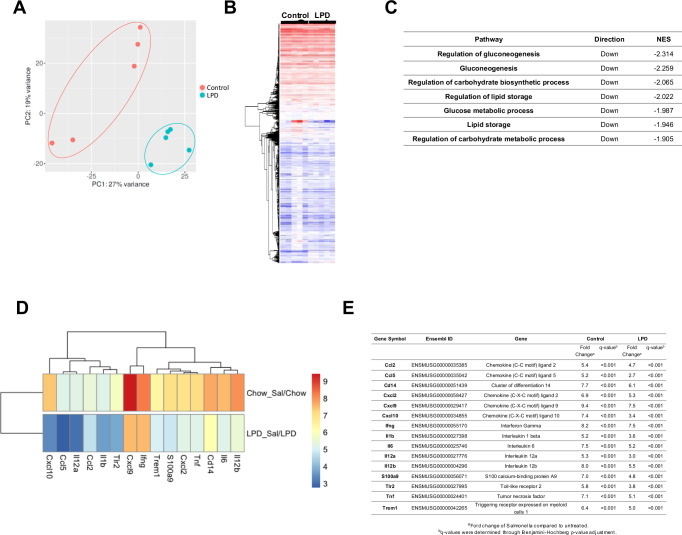
Bulk RNA sequencing of liver samples showing diet induced metabolic reprogramming and anti-inflammatory changes. **A** Principal component analysis (PCA) plot on raw gene count data for first and second component in normal and LPD liver samples infected with S. Typhimurium. **B** Heatmap of raw gene count data between treatment groups; Control vs LPD. **C** Table including pathway analysis using Gene Ontology (GO) database. the normalised enrichment score (NES) is generated from this analysis and indicates the distribution of gene ontology categories/gene sets across a list of ranked genes; a positive NES indicates an increase in the gene set, and a negative NES represents a decrease in the gene set. **D** Heat map showing fold change (Log2FC) of immune-related genes in normal and LPD and (**E**) associated statistical analyses of the genes shown. All *q*-value were <0.001; *q*-values were determined through Benjamini-Hochberg *p*-value adjustment.

To better understand the molecular changes in livers from normal diet- and LPD-fed animals after *S*. Typhimurium infection we performed bulk RNA-seq on whole liver lysates. The liver transcriptional profile of infected mice on the LPD clustered distinctly compared to that of infected mice on a normal diet by PCA (Fig. 2A) and the heatmap (Fig. 2B). The gene expression differences we observed in the control group may reflect biological variation in response to infection, while the control and LPD samples clusters were separated by PC1, constituting 29% of variation observed in data. The further analysis of differentially expressed genes (FDR, *q* < 0.001) between control and LPD diets after Salmonella infection showed: 2470 up-regulated genes and 2026 down-regulated genes in mice fed with a control diet, while LPD feeding led to 2301 up-regulated and 1860 down-regulated genes after S. Typhimurium infection.

From the pathway analysis, LPD induced molecular changes in liver cells compared to normal diet in mice after infection indicated by the down-regulation of key metabolic pathways including gluconeogenesis, lipid metabolism and OXPHOS-related processes (Fig. 2C).

Further fold change (Log2FC) analysis of gene expression, as shown in a heat map (Fig. 2D), confirmed the upregulation of genes in control vs LPD-fed mice after *S*. Typhimurium infection that included a number of proinflammatory genes *Cxcl2, Tnf, Tlr2, Nlrp3, Il1b, Il6, Il12a*, supporting the immunoregulatory effect of LPD (Fig. 2D). A table including the associated statistical analyses of the genes shown in Fig. 2E.

Together, our results point to metabolic rewiring and anti-inflammatory effects of LPD on the liver upon *S*. Typhimurium infection.

### Single cells analysis of immune cells in the liver captures reprogramming of monocytes

Following our RNA sequencing results suggesting the modulation of the innate immune response in LPD mice fed during infection, we performed single cell RNA sequencing using the 10x Chromium platform to further identify changes in cellular composition and gene expression programs specifically in innate immune cells.

**Fig. 3:**
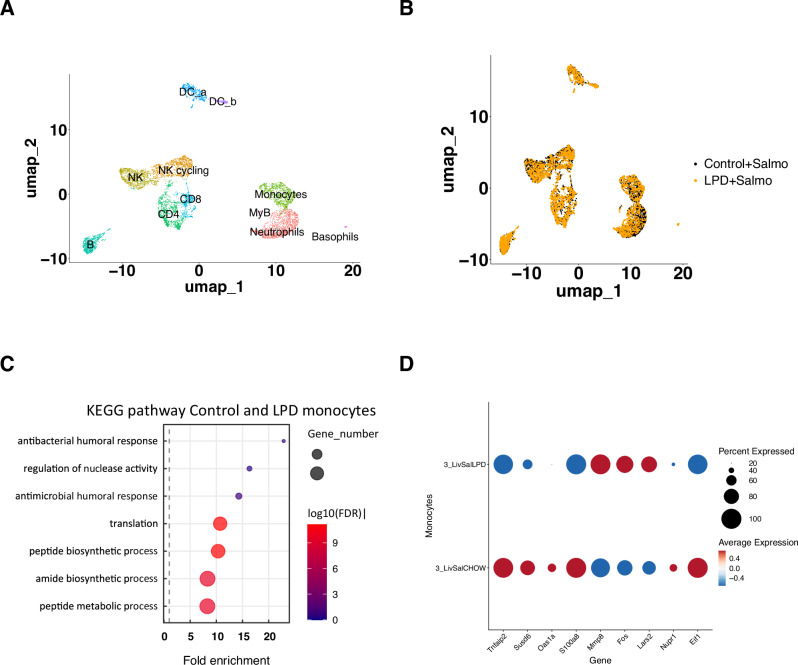
Single cell RNA sequencing on immune cells isolated from livers after infection. **A** UMAP representing cell types present among liver immune cells in normal and LPD fed animals after *S*. Typhimurium infection. **B** UMAP representing the cell type distribution in samples from animals fed normal and LPD diets. **C** KEGG analysis of pathways enriched in normal and LPD fed diets highlighting the metabolic remodelling of monocytes and enrichment for innate immune response genes in LPD-derived monocytes. **D** Dot plot representing differentially expressed genes between monocytes from normal and LPD diet calculated using differential expression (DE) testing based on the non-parametric Wilcoxon rank sum test included in Seurat package and Bonferroni correction for multiple testing^34^.

We isolated immune cells from livers^33^, followed by FACS purification for viable, CD45^+^ cells from control (*n* = 3) and LPD (*n* = 3) mice 3 days post *S*. Typhimurium infection (Supplementtary Fig. 1A). We generated standard 10 × 3’ scRNA-seq libraries from FACS-purified cell populations from control and LPD samples after infection (Fig. 3A).

In the control and LPD samples we recovered 3531 and 3611 cells respectively (Supplementary Fig. 2A). At first, we did not observe differences in the cell type distribution (Fig. 3B); however, more systematic quantification led to the observation of increased proportion of myeloid cells at the expense of lymphoid (Supplementary Fig. 2B). Further analysis identified 11 clusters within CD45^+^ liver cell population, which can be manually annotated using RNA marker expression in Seurat^34^ (Supplementary Fig. 2C). Since in our model we exposed animals to a short-term infection (3 days), we focused our analysis on cells of the innate immune response able to quickly respond to the pathogen-monocytes and macrophages^35^.

Differential gene expression analysis in control and LPD liver monocytes after *S*. Typhimurium revealed enrichment for nuclease activity, humoral and antibacterial response in normal diet compared to LPD monocytes after infection (Fig. 3C). We detected 53 downregulated and 21 upregulated genes in monocytes (Fig. 3D). Thus, after infection, monocytes from LPD-fed mice had decreased levels of *Tnfaip2* (Fig. 3D), whose expression is regulated by TNFα and other proinflammatory stimuli like IL1β and LPS via NFκB activation^36^. Our results also showed a reduction of *Susd6* (Fig. 3D) that promotes chemokine expression^37^. Furthermore, liver monocytes from LPD-fed mice expressed decreased levels of *Oas1a* (Fig. 3D), which stimulates the expression of chemokines (Ccl2, Ccl3, Ccl4, Ccl8, Cxcl9 and Cxcl10) upon inflammation in macrophages^38^. Additionally, LPD liver-monocytes expressed decreased levels of *S100a8* (Fig. 3D), which increases recruitment of leucocytes producing proinflammatory cytokines^39^. That was in line with the decreased frequency of B cells and CD3+ T cells we observed in our 10x data set (Supplementary Fig. 1B). Importantly, liver monocytes from LPD-fed mice expressed increased levels of *Mmp8* (Fig. 3D), a known inhibitor of macrophage *Mip-1α*, which drives the acute lung inflammation in mice^40^. Monocytes from infected mice fed with LPD expressed higher *c-Fos* levels compared to control diet-fed animals (Fig. 3D), a transcription factor involved in modulation of inflammation and decreased susceptibility to infection^41,42^.

In the LPD-fed infected with *S*. Typhimurium mouse samples, monocytes expressed lower levels of stress-induced transcription factor *Nupr1* (Fig. 3D), which activates mTOR pathway^43^ compared to normal-diet fed mice after infection. In line with this, we also found decreased expression of EIf1 that forms the 48S complex with mTOR effector Eif2 mediating its activation^44,45^, in liver monocytes from LPD-fed mice after Salmonella.

Overall, our combined bulk and single-cell RNA sequencing results show that while inducing myeloid cell expansion, LPD diet simultaneously reprogrammed these cells metabolically, including modulating mTOR, rendering them anti-inflammatory and likely more efficient at bacterial clearance.

### Low amino acid availability decreases the expression of inflammasome components, proinflammatory cytokines and mTOR activation in mouse bone marrow derived macrophages

Nutrient sensing pathway-mTOR is the key regulator of monocyte/macrophage response to infection by promoting inflammasome activation and thus production of proinflammatory cytokine IL1β, while restricting the activation of autophagy^46^. mTOR is regulated by amino acid availability^47^ and this balance may mediate the effect of the LPD on the macrophage activation we observed in response to Salmonella infection in vivo.

**Fig. 4:**
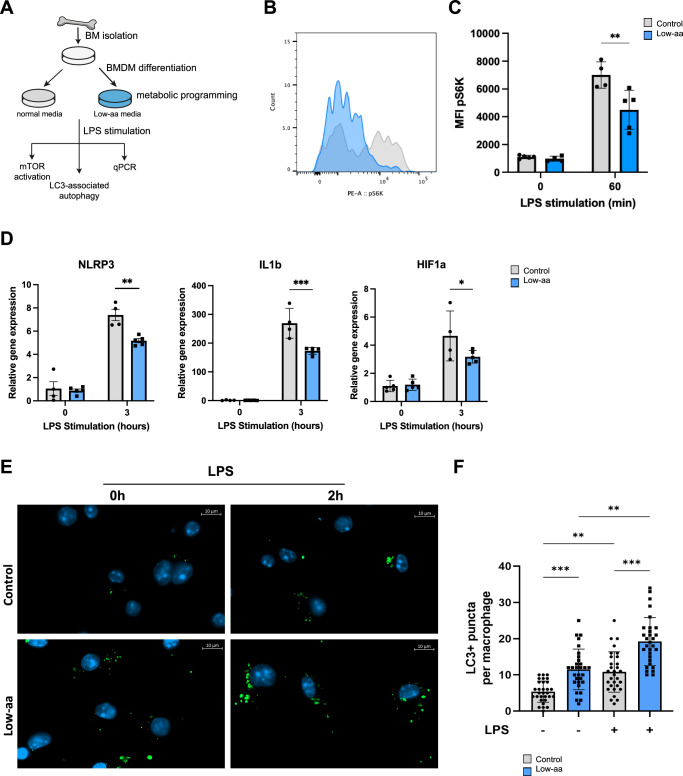
Low-aa culture media inhibits mTOR and activates LC-3 dependent autophagy in BMDM in response to LPS in vitro. **A** Experimental set up. **B** Representative histogram depicting decreased MFI of pS6K kinase level in Low-aa media programmed BMDM’s compared to normal media. **C** Quantification of pS6 kinase level upon LPS treatment of BMDM’s from normal or Low-aa media, applied two-way ANOVA with Sidak’s multiple comparisons test, *p* = 0.0015. **D** qPCR expression analysis of Nlrp3, Il1b and HIF1a; Ctrl vs Low-aa after LPS *p* = 0.0014, *p* < 0.0001, *p* = 0.0467 respectively, Two-Way ANOVA with Sidak’s multiple comparisons in BMDM. **E** Representative images of immunofluorescence staining for LC3 and further (**F**) quantification in control and low-aa media; Ctrl vs Low-aa *p* < 0.001, Ctrl vs Ctrl+LPS *p* = 0.003, Low-aa vs Low-aa+LPS *p* < 0.002, Ctrl+LPS vs Low-aa+LPS *p* < 0.001, Kruskal–Wallis test. Representative images are shown from 63x magnification. In vitro experiments were repeated 2-3x with *n* = 3–4 replicates. Values are mean ± SEM.

To test the hypothesis that changes in amino acid availability affect macrophage activation, we performed in vitro experiments using bone marrow derived macrophages (BMDMs) that we cultured in a low amino acid media (herein Low-aa) to mimic LPD condition in vivo. As a model of infection, we stimulated BMDM cultured in control or Low-aa media with lipopolysaccharide (LPS) for the indicated times (Fig. 4A). To measure the activation of the mTOR pathway we performed intracellular FACS staining for phosphorylated S6 kinase (pS6K, Ser 235, 236), which activates protein translation^48,49^ and observed 1.5-fold increased levels of pS6K in control compared to Low-aa BMDMs (Fig. 4B, C), confirming reduced mTOR activation in the latter. To study the effect of decreased mTOR pathway activation on the expression of inflammation-propagating targets, we determined the levels of the inflammasome subunit Nlrp3, which was 2-times lower expressed in Low-aa BMDMs compared to control after LPS stimulation. Reduced Nlrp3 levels correlated with decreased expression of IL1β (Fig. 4D) and decreased Hif1α expression (Fig. 4D) after LPS, supporting the reduced activation of macrophages when cultured in a Low-aa media.

The cross-regulation between inflammasome and autophagy to control the inflammatory response is well established^46^. Thus, we next evaluated the level of autophagy in BMDMs exposed to Low-aa media by detecting LC3B puncta, a surrogate of autophagy. Immunofluorescence staining of LC3B puncta and its further quantification in single macrophages revealed 2-fold increased autophagy activity in BMDMs cultured in Low-aa compared to control media in response to LPS (Fig. 4E, F).

Together these results show that Low-aa availability, consistent with a low protein diet feeding in vivo, reprograms BMDMs to become less proinflammatory while increasing autophagy.

### Restoration of mTOR activation with dietary leucine supplementation abolishes the LPD-mediated protective effects on liver function via regulating macrophage phagocytosis and autophagy

**Fig. 5:**
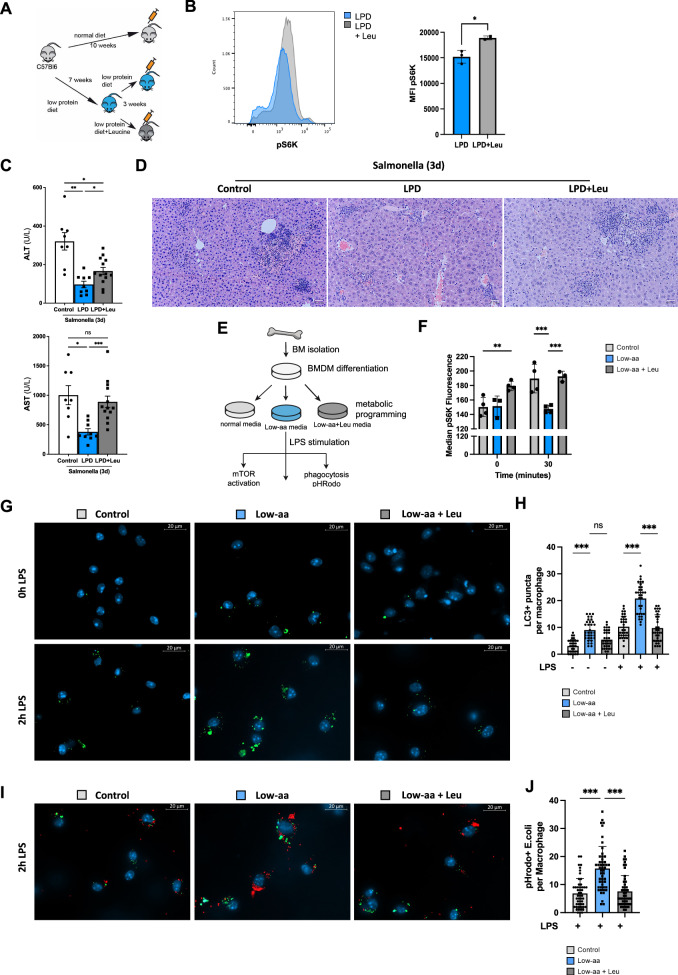
Supplementation with leucine diminishes LPD-mediated protection from liver damage upon *S.* Typhimurium infection via BMDM metabolic reprogramming. **A** In vivo experimental set up. **B** Intracellular FACS staining for pS6 kinase in primary bone marrow F4/80+ macrophages stimulated ex vivo 60 min with LPS and quantification of pS6 kinase MFI, unpaired Student t-test, two-tailed, *p* = 0.03. **C** Serum level of ALT (Ctrl vs LPD *p* = 0.028; LPD vs Leu+LPD *p* = 0.181; Ctrl vs Leu+LPD *p* = 0.030, Brown-Forsythe and Welch ANOVA test) and AST (Ctrl vs LPD *p* = 0.0122; Ctrl vs Leu+LPD n.s.; LPD vs Leu+LPD *p* = 0.0005, Brown-Forsythe and Welch ANOVA test) and (**D**) H&E staining on liver sections from LPD and LPD+Leu fed mice, 3 days after *S*. Typhimurium. **E** In vitro experimental set up for BMDM. **F** Quantification of intracellular FACS staining for pS6 kinase in BMDM with control, Low-aa or Low-aa+Leu media, Non-stimulated: Ctrl vs Low-aa+Leu *p* = 0.01; 30 min after LPS: Ctrl vs Low-aa *p* < 0.001, Low-aa vs Low-aa+Leu *p* < 0.001, One-Way ANOVA with Tukey’s multiple comparisons test. **G** Immunofluorescence (IF) staining for LC3 puncta and (**H**) further quantification of per BMDM; Ctrl vs Low-aa *p* < 0.001, Low-aa vs Low-aa+Leu n.s., Ctrl+LPS vs Low-aa+LPS p < 0.001, Low-aa+LPS vs Low-aa+Leu+LPS *p* < 0.001, Kruskal–Wallis test. **I** IF for LC3 (green) and pHrodo E. coli beads (red) and (**J**) further quantification ***p < 0.001 at 2 h after LPS. **H**, **J** One-Way ANOVA, Kruskal–Wallis test with Dunns correction Results shown are representative from 2 independent experiments. Representative microscopic images are shown from 63x magnification. Values are mean ± SEM.

Next, we aimed to assess the physiological impact of metabolic reprogramming in macrophages via the re-activation of mTOR in response to infection in vivo. To do this, we supplemented the LPD with leucine, an essential amino acid well-characterised as an activator of the mTOR pathway^50^. A group of mice was fed with a LPD diet for 7 weeks and then switched to LPD diet supplemented with 3% leucine (LPD+Leu), while other groups were fed with a control diet or a LPD for the duration of the experiment (10 weeks) (Fig. 5A).

To measure the mTOR activation in primary bone marrow F4/80^+^ macrophages^51^ cells were FACS-sorted from LPD or LPD+Leu fed animals (Supplementary Fig. 3A). Isolated cells were stimulated with LPS (100 ng/ml) for 60 min prior to fixation, permeabilization, staining and FACS analysis of mTOR activation. We observed 2-fold increased pS6K level in LPD+Leu cells compared to LPD in purified F4/80^+^ cells, supporting the restoration of mTOR activation in macrophages in response to leucine supplementation of LPD in vivo (Fig. 5B). This observation points that a LPD modulates macrophage mTOR activation in the bone marrow.

In this context, we determined the effects of dietary leucine restoration in the liver response to *S*. Typhimurium infection. Analysis of the liver enzymes AST and ALT in serum showed a significant increase in the LPD+Leu group compared to LPD, pointing to a worsening in liver damage upon leucine supplementation (Fig. 5C). When compared to control diet-fed mice, the addition of LPD with leucine restored AST levels to comparable levels to control diet fed mice, while ALT was still significantly lower than in control (Fig. 5C). Histological analysis of liver sections confirmed the increased liver injury in control and LPD+Leu mice that presented larger areas of necrosis compared to LPD fed mice after *S*. Typhimurium infection (Fig. 5D).

To further investigate the mechanisms mediating the loss of the protective effects of a LPD against infection after leucine supplementation in vivo, we performed in vitro experiments in BMDM cultured in a Low-aa media supplemented with leucine (Low-aa+Leu) and stimulated with LPS (Fig. 5E). Following our observations in vivo in macrophages isolated from the BM of LPD+Leu mice, the analysis of mTOR pathway activation confirmed increased S6 kinase phosphorylation in BMDM cells cultured in Low-aa+Leu media compared to control and Low-aa media at basal conditions. S6 kinase phosphorylation was further increased upon LPS stimulation, while it remained unaffected in BMDM on Low-aa media (Fig. 5F).

To evaluate the level of autophagy, BMDM were stimulated with LPS for 2 h, fixed, permeabilized and stained with LC3B. Quantification of the number of LC3B puncta was highest in Low-aa media-BMDM and the lowest in control cells (Fig. 5G). Macrophages programmed with Low-aa+Leu showed increased number of LC3B puncta compared to control, however this was still lower than Low-aa media, suggesting partial reduction of the Low-aa induced autophagy activation (Fig. 5H).

Autophagy and phagocytosis are important mechanisms regulating innate immunity. Phagocytic potential of macrophages was measured using E. coli pH Rodo bioparticles^52^. We did not observe differences in non-stimulated cells cultured with the different media, however after LPS stimulation we found a significant increase in the number of bioparticles per macrophage in Low-aa programmed cells compared to control or Low-aa+Leu cells (Fig. 5I). The Low-aa+Leu phagocytic cell potential resembled the pattern observed in control macrophages, where up to 70% of cells have 0-5 bioparticles/cell and Low-aa+Leu had 60% while in Low-aa programmed BMDM this constituted only 30% of the cells. The Low-aa+Leu phagocytic cell potential resembled the pattern observed in control macrophages (average 7 or 6 particles/macrophae with a range 0-20), while it was 2 times higher in Low-aa media (with a range of 2-38 particles/macrophage) (Fig. 5J). Finally, colocalization of red bioparticles with stained LC3B was especially striking in Low-aa programmed macrophages suggesting that Low-aa programming likely increases the phagocytic potential of BMDM (Fig. 5I, J), supporting that this process depends on the balance between mTOR activation, autophagy and phagocytosis^21^.

Next, we wanted to determine whether these interventions restricting protein/amino acid content could modulate the phagocytic potential and autophagy also in human macrophages.

We isolated peripheral blood mononuclear cells (PBMC) from healthy individuals and differentiated them into macrophages (PBMC-derived macrophages), as we previously described^53^. Fully differentiated macrophages were plated in control, Low-aa or Low-aa+Leu media (Supplementary Fig. 4A). FACS analysis of pS6K level showed 2–3 fold increase in control and Low-aa+Leu macrophages compared to Low-aa, indicating decreased mTOR activation in Low-aa PBMC-derived macrophages (Supplementary Fig. 4B, C). Next, we determined the autophagic potential of human PBMC-derived macrophages cultured in the different media. Similarly, to our observations in mouse BMDM, PBMC-derived macrophages cultured in Low-aa media had a 2-fold increased number of LC3B puncta per macrophage compared to the control and Low-aa+Leu, pointing to an increased autophagy level (Supplementary Fig. 4D, E). To evaluate if also the phagocytic potential of PBMC-derived macrophages was altered in control, Low-aa and Low-aa+Leu culture conditions, we analysed the phagocytosis of pHRodo bioparticles. Here, again we observed that human PBMCs-derived macrophages cultured in Low-aa media had a 1.5-2-fold increased number of bioparticles compared to the control or Low-aa+Leu media (Supplementary Fig. 4F, G), following our observations in murine BMDM (Fig. 5).

Together, culture conditions resembling LPD reprogrammed human PBMC-derived macrophages to decrease activation of mTOR and increase phagocytic potential suggesting that the mechanism is conserved in mouse and human macrophages.

Overall, amino acid stress imposed by a Low-aa shifts the balance from mTOR activation, inducing expression of proinflammatory cytokines, towards increased autophagy and bacteria phagocytosis that was reverted after mTOR restoration with Leucine supplementation. Importantly, here we demonstrate how changes in dietary protein and specific amino acid content modulate the innate immune response to infection.

## Discussion

Predominantly in western countries, dietary habits are increasingly shifting towards healthier choices including the reduction of meat (hence dietary protein) intake. Still, the impact of these dietary changes, particularly in the immune response, remain largely undefined.

Previous studies have demonstrated that drastic reduction of protein content (0.5–2.5% protein) during malnutrition severely impairs immune system function and increases susceptibility to infections^54^. In our study, we have analysed the effects of a reduction in the protein intake (6% low protein diet; LPD), rather than malnutrition, and identified a protective effect of a LPD in adult mice, as supported by the preserved liver parenchymal status in response to *S*. Typhimurium infection.

To better understand the mechanisms mediating the protective effects observed in LPD-fed mice in response to infection, we performed bulk RNA sequencing of whole liver tissue followed by single-cell RNA sequencing that allowed us to characterise the liver phenotypic changes, as well as to pinpoint the key contributing immune cells.

Our bulk RNA-sequencing analysis of liver lysates revealed significant metabolic changes that associated with decreased expression of proinflammatory cytokines, inflammasome subunits, and pattern recognition receptors in LPD-fed mice compared to normal-fed mice after *S*. Typhimurium infection. These results support the immunoregulatory rewiring effects of the LPD intervention in response to infection, which is associated with protection from S. Typhimurium-induced liver injury and pointed to macrophages as potential mediators of the effects observed.

Macrophages are a key component of the host innate immune defence against *S*. Typhimurium infection via pathogen phagocytosis and production of proinflammatory cytokines (i.e. IL1β)^55^. Previous studies have shown that malnutrition decreases macrophages phagocytosis and killing capacity, as well as reduces adhesion, spreading, and anti-fungicidal activity supporting that severe malnutrition significantly impairs macrophage function and diminishes response to acute and chronic infections^56,57^. In contrast, our bulk RNA sequencing results pointed to changes in macrophage activity, an anti-inflammatory phenotype and a more effective response to bacterial infection in LPD-fed mice.

To better define the cellular and molecular changes occurring in myeloid cells, we next performed a single cell RNA sequencing. The analysis of transcriptionally defined cell types revealed the increased fraction of myeloid cells (monocytes and neutrophils) at the expense of lymphoid cells, supporting the relevance of these cells as mediators of the differential outcomes observed in control and LPD-fed mice. Intriguingly, despite their expansion, these myeloid cells underwent substantial metabolic reprogramming and acquisition of an anti-inflammatory phenotype that associated with an enhanced efficacy in responding to Salmonella infection in LPD-fed mice. Hence, we focused our downstream analysis on monocytes, which give rise to macrophages^58^.

Detailed analyses of cell-specific genetic changes using single cell RNA-sequencing approach revealed decreased expression of key markers of macrophage activation such as S100a8, which promotes the recruitment of leucocytes and a proinflammatory cytokine milieu^39^. In murine models the blockade of the S100a8/S100a9 complex with small molecules or antibodies improves pathological conditions, while decreased expression of this complex correlates with better prognosis, as sepsis surviving patients shown decreased S100A8/A9 levels compared with non-survivors^59^. Targeting S100A8/A9 can also prevent liver injury as well as bacterial dissemination at an early phase during human sepsis and endotoxemia^60^.

We observed that the protection against *S*. Typhimurium infection after LPD also correlated with a significant increase in c-Fos expression in monocytes. This finding aligns with recent studies, where deletion of *c-Fos* in mouse monocyte and macrophages led to significantly enhanced production of TNFα, IL6 and IL12 p40 in response to LPS and an increased susceptibility to *S*. Typhimurium infection^41,42^.

Interestingly, gene expression changes in monocytes were not restricted to inflammation but we also found changes of key metabolic factors in infected mice fed with LPD compared to control diet, including a decrease of Nupr1 and Eif1, both mediators of mTOR activation^43,44^, in monocyte/macrophages suggesting decreased activity of mTOR pathway while enhanced autophagy in these cells after LPD feeding. The coordinated expression pattern of proinflammatory and mTOR-related genes strongly suggests mTOR role as a molecular switch and transcription factor executing nutritional and molecular programs. ChIP-sequencing datasets revealed that mTOR directly binds to thousands of regulatory regions of polymerase II-transcribed genes in both mouse liver and human prostate cancer cells^61,62^. Interestingly, treatment of prostate cancer cells with the inhibitor of polymerase II transcriptional activity α-amanitin, which has no effect on classic cytoplasmic mTOR-regulated signalling pathways-autophagy and phagocytosis, abrogated the metabolic reprogramming associated with the transcriptional function of nuclear mTOR observed in these cells^61^. Thus, future studies will be necessary to unravel mTOR function as transcription factor in response to a range of diets fed and at different developmental stages.

mTOR is a known regulator of macrophage activation by inhibiting autophagy^63^, while autophagy is essential to control the host response to pathogens^64^ via modulation of the inflammasome and IL1β production^65^. Hence, impaired autophagy enhances the inflammasome activity and IL1β production in macrophages after LPS^66^. Thus, the immunomodulation and metabolic rewiring in monocytes upon LPD we observe could lead to improved ability to resolve *S*. Typhimurium infection and thus restricting liver tissue damage via modulating the mTOR/autophagy axis. To test this and define the regulation of the mTOR/autophagy as a mechanistic mediator of the protective effects against infection we observed upon LPD feeding in vivo, we performed studies in vitro using mouse BMDM. Our results show that low amino acid content in culture media, mimicking low protein feeding in vivo, reduced macrophage proinflammatory nature and concurrently enhancing autophagy. Our results showing that reduced availability of amino acids in culture led to significant reduction of inflammasome-IL1β-Hif1α are in line with previous reports showing Hif1α-induced activation of the inflammasome, IL1β production and impaired autophagy flux in macrophages in patients with chronic liver inflammation during MASLD^67^.

Ultimately, to pinpoint the attenuation of mTOR signalling as the mechanistic mediator of the protective effects of LPD during infection we restored mTOR signalling in LPD fed mice in vivo by dietary supplementation of leucine; an essential amino acid that directly activates the mTORC1 subunit of the mTOR complex^50^. In agreement with our hypothesis, leucine supplementation reverted the protective effects of a LPD in response to *S*. Typhimurium infection in vivo. The direct activation of mTOR by leucine-supplementation in cultured BMDM diminished the autophagy and phagocytic potential of these cells supporting the crucial immunomodulatory role of dietary amino acids, and more specifically of leucine. These observations were resembled in human PBMC-derived macrophages metabolically programmed as mouse cells, hence suggesting that this mechanism is applicable to mice and humans. Our observations in human PBMC-derived macrophages may be relevant for human health. Thus, previous studies showed how the consumption of leucin-rich diets (western diets), which contributed to mTOR activation, associated with obesity^68^, a condition characterised by dysfunction of innate immune response^69^ and increased risk for bacteraemia^70^.

Overall, our findings highlight the prospects to improve the immune response to infection using dietary interventions, and more specifically that dietary interventions involving the reduction of leucine may pose beneficial potential to boost innate immunity. Still, to fully utilise these benefits, future work is guaranteed to better understand the kinetics of metabolic changes induced by LPD to choose the most optimal age/time window.

## Methods

### Experimental procedures in animals

C57BL/6J mice (CD45.2), were purchased (Charles River Massachusetts, United States) and housed at the Disease Modelling Unit (University of East Anglia, Norwich, United Kingdom). We have complied with all relevant ethical regulation for animal use. All experiments were approved by the Animal Welfare and Ethical Review Body (University of East Anglia, Norich, UK) and the Home Office (PPL: 9417531 to Beraza). All procedures were carried out following the guidelines of the National Academy of Sciences (National Institutes of Health, publication 86-23, revised 1985) and were performed within the provisions of the Animals (Scientific Procedures) Act 1986. Mice were kept in individually ventilated cages and housed under specific pathogen-free conditions in a 12/12-h light/dark cycle. Animals were put on *ad libitum*, isocaloric control (Special Diets Services, 801066) or 6% low protein diet (Teklad, TD220065) for 10 weeks, and body weight was regularly monitored. Low protein diet with 3% leucine was purchased from Teklad (TD.90016). Mice used were 8-10 weeks old, male animals were used. For the differentiation of bone marrow derived macrophages mice were exposed to CO2, BM was harvested for in vitro differentiation.

For human PBMCs isolation and culture, leucocyte cones were obtained from NHS Blood and Transplant (NC24) that provides appropriately consented materials to approved recipients for approved non-clinical uses through a managed, governed service called “Non-clinical Issue” (NCI). The use of these samples was approved by the Faculty of Medicine and Health Sciences Research Ethics Committee (University of East Anglia) approval 2019/20-097 and NHS Blood and Transplant approval R347. Collection and handling of human samples used in this study conformed to the Declaration of Helsinki and the Human Tissue Act (UK) and Good Clinical Practice Guidelines (UK).

### *S*. Typhimurium infection model

Glycerol stock of Salmonella *enterica* serovar Typhimurium (*S*. Typhimurium) (SL1344- JH3009) was plated on Luria Broth agar plates and the colonies were inoculated and grown overnight into 5 ml of Luria Broth with 0.3 M NaCl (LBS). The overnight culture was then diluted 1:100 in LBS and grown until the culture optical density (ΔOD600nm) of 1.2–1.4 (late exponential phase). This is the time point where SPI1 invasion genes are turned on in *S*. Typhimurium. The bacterial culture was then centrifuged at 3000 × *g* for 7 min before washing bacterial cells twice in 25 ml of sterile DPBS at room temperature. Finally, bacterial cells were resuspended in sterile DPBS at a concentration of (1–5) × 10^8^ CFU per 100 μl of DPBS (knowing that OD600nm 1.26 corresponds to 7.53 × 10^8^ CFU/ml). Mice were infected with 100 μl of 1 × 10^8^ CFU *S*. Typhimurium (SL1344-JH3009) by intraperitoneal injection for 3 days. The mice were anaesthetised using isoflurane to collect blood, followed by exposure to CO_2_, and the liver was collected for flow cytometric analysis, sorting and sequencing.

### Liver histology

Liver tissues were harvested and immediately fixed in 10% neutral formalin and embedded in paraffin blocks 24 h later. Tissue blocks were sectioned, dewaxed, and hydrated prior to being stained with Hematoxylin & Eosin (H&E) for histopathological analysis. Slides were imaged using brightfield on a BX53 upright microscope (Olympus) with an Olympus DP74 colour camera and a pT100 LED transmitted light source (CoolLED).

### Serum transaminases

The levels of circulating ALT and AST were measured in serum samples in a Randox RX Daytona analyser.

### Macrophage differentiation and culture

For mouse, BMDMs were differentiated from bone marrow cells isolated from WT mice. The femur and tibia were cut in the middle and placed in a 0.5 ml Eppendorf tube in which a hole was made to allow the removal of the BM, placed in an intact 1.5 ml Eppendorf and centrifuged 1000 × *g* for 6 s to collect the BM cells. The BM pellet from each mouse was pooled and plated with RPMI-1640 (Gibco, Thermo Fisher Scientific) supplemented with 20% foetal bovine serum (FBS) (Gibco, Thermo Fisher Scientific), 30% L929 conditioned media and 1% penicillin/streptomycin (Gibco, Thermo Fisher Scientific).

For human PBMCs isolation and culture, leucocyte cones were obtained from NHS Blood and Transplant (NC24) under Faculty of Medicine and Health Sciences Research Ethics Committee approval 2019/20-097 and NHS Blood and Transplant approval R347. Blood was drained from the cones and made up to 40 ml with PBS lacking calcium and magnesium (Gibco, Thermo Fisher Scientific). Blood was layered on top of 2 × 20 ml Ficoll-Paque PLUS (Cytiva) and centrifuged at 400 × *g* for 35 min (acceleration 5, deceleration 0) before the interface layer of peripheral blood mononuclear cells was isolated and made up to 40 ml with HBSS lacking calcium and magnesium (Gibco, Thermo Fisher Scientific). Cells were centrifuged at 80 × *g* for 10 min, supernatants discarded, and pellets resuspended in HBSS before the centrifugation step was repeated to wash the cells. Pellets were resuspended in RPMI-1640 and diluted to 20 × 10^6^ cells/ml before being layered on top of 2 × 20 ml Percoll solution [45% Percoll PLUS (Cytiva 11500744), 10% 10x PBS]. Cells were centrifuged at 400 × *g* for 30 min (acceleration 0, deceleration 0) and the interface layer was isolated and made up to 50 ml with RPMI-1640. Cells were centrifuged at 80 × *g* for 10 min and resuspended in RPMI-1640 to a density of 2 × 10^6^/ml before being plated in 10 cm tissue culture plates and incubated (37 °C, 5% CO_2_) for 60 min to allow highly adherent cells to attach to the plate. Medium containing any non-adherent cells (mainly lymphocytes and neutrophils) was removed and fresh RPMI-1640 was added supplemented with 5% FBS (Gibco, Thermo Fisher Scientific), recombinant human M-CSF (100 ng/ml, Peprotech 300-25), and 1% penicillin/streptomycin before being incubated for 7 days.

Cells were allowed to differentiate into macrophages for 7 days, with fresh media added on day 3. A total of 1 × 10e6 adherent cells were then plated for experiments in 6-well plates and 500k for 12-well plates. Human macrophages were dissociated with 0.02% EDTA solution (Merck E8008) and removed from the plates with a cell lifter (Corning CLS3008-100EA), whilst mouse macrophages were dissociated using ice-cold PBS. For immunofluorescence imaging, cells were plated on glass coverslips.

Macrophages were then cultured in DMEM-LM medium (Thermo scientific), normalised to 1.4 g/l glucose in DPBS (Gibco, ThermoFisher Scientific), supplemented with 3% L929 conditioned media for mice or 10 ng/ml M-CSF for human, and 1% penicillin/streptomycin. To replicate normal diet, 10% FBS and 1 X MEM amino acid solution (Gibco, ThermoFisher Scientific) were also added to the medium, and to replicate LPD diet in mice, a Low amino acid media (Low-aa) was created by adding 1% FBS and 0.2 X MEM amino acid solution (adjusted for the leucine concentration) for 48 h. To assess the effect of leucine in Low-aa medium, 1965mg/L leucine (equivalent to leucine concentration in MEM) was added to Low-aa medium. BMDMs were then starved by removing FBS from the mediums for 12 h prior to 100 ng/mL LPS stimulation.

### Assessment of LC3-associated autophagy and phagocytosis

For determination of autophagy, the metabolically reprogrammed macrophages were treated with 100 ng/ml LPS for 2 h. For determination of phagocytic potential, cells were treated with 100 ng/ml LPS and 10ug/ml pHrodo^TM^ Red E.coli BioParticles^TM^ Conjugate for Phagocytosis (ThermoFisher Scientific) for 2 h. Cells were stimulated with 100 ng/ml LPS for 2 h. Cells were then fixed using 4% formaldehyde solution, buffered pH 6.9 (Sigma-Aldrich) and permeabilised using solution B from FIX & PERM^TM^ Cell Permeabilization Kit (Invitrogen^TM^, ThermoFisher scientific), during which cells were stained with 1:300 dilution of LC3-FITC antibody (EPR18709, Abcam). Cells were washed and mounted using Vectashield Antifade mounting medium with DAPI (Vector Labs) imaged using AxioImager M2 (Zeiss) using x63 magnification and oil immersion. The number of LC3-positive puncta and pHrodo^TM^ E.coli BioParticles^TM^ per macrophage were quantified using Fiji Image J (2.9.0/1.53t).

### Quantitative real-time PCR

RNA from cells was isolated using the ReliaPrep RNA Miniprep System (Promega) according to manufacturer’s instructions and RNA quality was checked using a NanoDrop spectrophotometer (Thermo Scientific). RNA was reverse transcribed using UltraScript^TM^ cDNA Synthesis Kit (PCR Biosystems) according to manufacturer’s instructions, with a total reaction volume of 10 ul, and a Thermocycler (Bio-Rad). A pre-defined program was run which consisted of 42 °C incubation for 30 min followed by an 85 °C incubation for 10 min. The reaction was held for 4 °C until samples were stored at −20 °C. qRT-PCR was then performed using qPCRBIO SyGreen Mix (PCR Biosystems), according to manufacturer’s instructions and a 5 ul total reaction volume in a clear 384 Well Style Optical qPCR Plate (Scientific Laboratory Supplies), sealed with Fisherbrand™ Adhesive Plate Seals (Fisher Scientific), and run on the QuantStudio 7Flex Real-Time PCR System (Applied BioSystems) using a pre-defined method of a 3 min hold stage at 95°C, a PCR stage consisting of 35 cycles of 15 s at 95°C, followed by 1 min at 60 °C, and then a melt-curve stage of 95 °C for 15 s, 60 °C for 1 min, and then 95 °C for another 15 s. The following primers were used: Tbp1 (forward: GAAGCTGCGGTACATTCCAG; Reverse: CCTTGTACCCTTCACCAAT) Interleukin-1β (QuantiTect Primer Assay Qiagen, GeneGlobe ID - QT01048355), HIF-1α (Forward: TCATCAGTTGCCACTTCCCC. Reverse: CCGTCATCTGTTAGCACCA) and NLRP3 (Forward: GGGCTTAGGTCCACACAGA. Reverse: ACACGAGTCCTGGTGACTT)

### Flow cytometry and sorting

For single cell RNA sequencing cells were stained with CD45-APC-Cy7, and DAPI, and sorted on or BD FACS Aria-Fusion to purify viable, CD45+ cells that were loaded on 10X.

For intracellular FACS staining metabolically reprogrammed macrophages were dissociated from cell culture plates and fixed using ice cold methanol, washed and stained with 0.1 uL pS6K, (cupk43k, eBioscience^TM^) per sample. A BD FACS Symphony A1 (Becton, Dickinson and Company), was used to assess pS6K expression and data was analysed using FlowJo 10.9.0 (Becton, Dickinson and Company).

### Sequencing of single-cell cDNA libraries

Sorted cells were processed by 3’ end single-cell RNA-Seq using the 10X Genomics Chromium (V3 Kit) according to the manufacturer’s protocol (10X Genomics, Pleasanton, CA). Libraries were sequenced on a NovaSeq 6000 (Illumina, San Diego) in paired-end, single index mode as per the 10X Genomics recommended metrics.

The single cell data was processed using 10X Genomics Cell Ranger analysis pipeline (cellranger-6.0.1) with Ensembl GRCm39 *Mus musculus* assembly and gene annotation. A feature barcode matrix was generated for each sample by applying the *cellranger count* pipeline. All feature barcode matrices were aggregated using *cellranger aggr*, which normalises sequencing depth across samples. QC was performed excluding cells with fewer than 1000 genes detected or more than 5% of UMI counts associated to mitochondrial genes. In total, 7142 cells were selected, distributed as follows: 3531 cells came from the immune liver cells S. Typhimurium treated Control diet sample and 3611 cells from the immune liver cells S. Typhimurium treated Low Protein diet sample. Cell cycle variation was removed using the ‘CellCycleScoring’ method followed by regressing out ‘S.Score’ and ‘G2M.Score’. Cells were then normalised to 10,000 UMIs per cell and logarithmically transformed. HVGs were selected using the ‘FindVariableFeatures’ method. UMAP visualisations were obtained from 20 PCA components, and clusters were defined at a resolution of 0.3 using Louvain algorithm. Cell types were annotated using typical marker genes for the different haematopoietic populations. Differential gene expression was performed using the ‘FindMarkers’ method in Seurat^34^.

### Statistics and reproducibility

Statistical analyses were performed using GraphPad Prism software version 10.0.3. Statistical differences between two groups were determined by unpaired, two-tailed Student’s *t* test with Welch’s correction.

For Bulk-RNAseq on liver tissues, *q*-values were determined through Benjamini–Hochberg *p*-value adjustment and *q*-value were <0.001. For single cell RNA-seq adjusted *p*-values were derived using non-parametric Wilcoxon rank sum test included in Seurat package and Bonferroni correction for multiple testing.

Two-way ANOVA tests were used when comparing different treatments at different timepoints and were performed using GraphPad Prism software settings. Data are shown as mean ± SEM.

Illustrations were generated using Adobe Illustrator and bioicons (https://bioicons.com/).

### Reporting summary

Further information on research design is available in the Nature Portfolio Reporting Summary linked to this article.

## Supplementary information

Supplementary Information

Reporting Summary

## Data Availability

Bulk RNA-seq and scRNA-seq data generated for this study have been deposited at ENA and are publicly accessible using accession number:PRJEB74911. The underlying raw data shown in Figs. 1–5 can be found in Supplemental Data 1. Additional Supplementary results are shown in Supplemental material and corresponding raw data can be found in Supplemental Data 2. The authors declare that all data generated from this study are available within the manuscript and the supplemental material provided. Any additional files or information can be provided upon request to the corresponding authors.
