# Supplementary material for: Low protein diet protects the liver from Salmonella Typhimurium-mediated injury by modulating the mTOR/autophagy axis in macrophages
Source: Commun Biol. 2024 Sep 30;7:1219. doi: 10.1038/s42003-024-06932-w (PMC11444042; doi:10.1038/s42003-024-06932-w)
Supplement: Supplementary file 1 — Supplementary Information [file 42003_2024_6932_MOESM1_ESM.pdf]

Low protein diet protects the liver from Salmonella Typhimurium-mediated injury by modulating the mTOR/autophagy axis in macrophages.

Edyta E Wojtowicz[1], Katherine Hampton[2], Mar Moreno-Gonzalez[3, 4,5], Charlotte L Utting[1], Yuxuan Lan[1], Paula Ruiz[3, 4,5], Gemma Beasy,[4] Caitlin Bone[3], Charlotte Hellmich[2, 6], Rebecca Maynard[2], Luke Acton[5], Matthew Markham[2], Linda Troeberg[2] Andrea Telatin[7], Robert A Kingsley[6,8], Iain C Macaulay[1], Stuart A Rushworth[2,9], Naiara Beraza[3, 4, 5, 9]

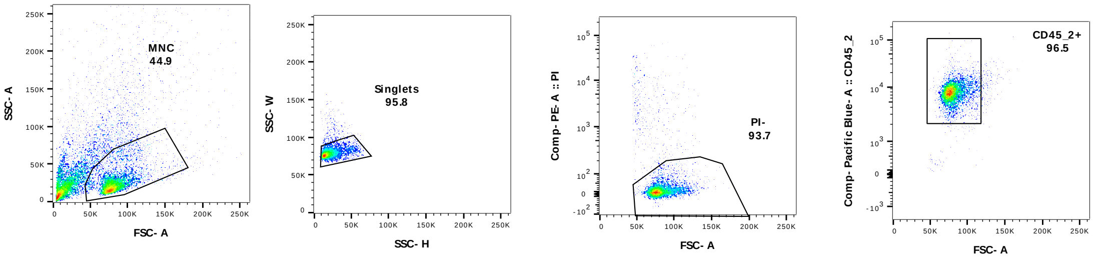

Supplementary Figure 1

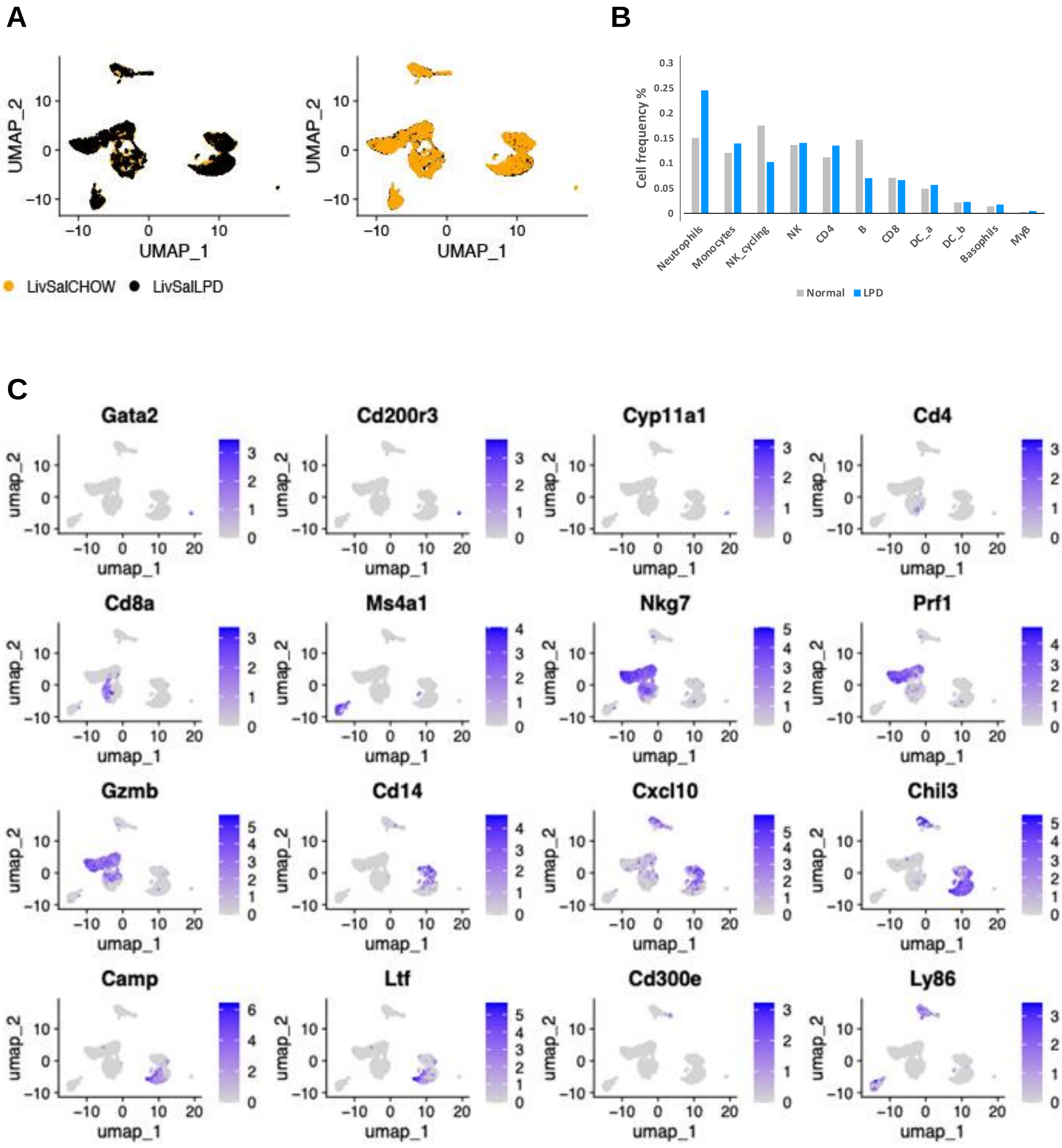

Supplementary Figure 2

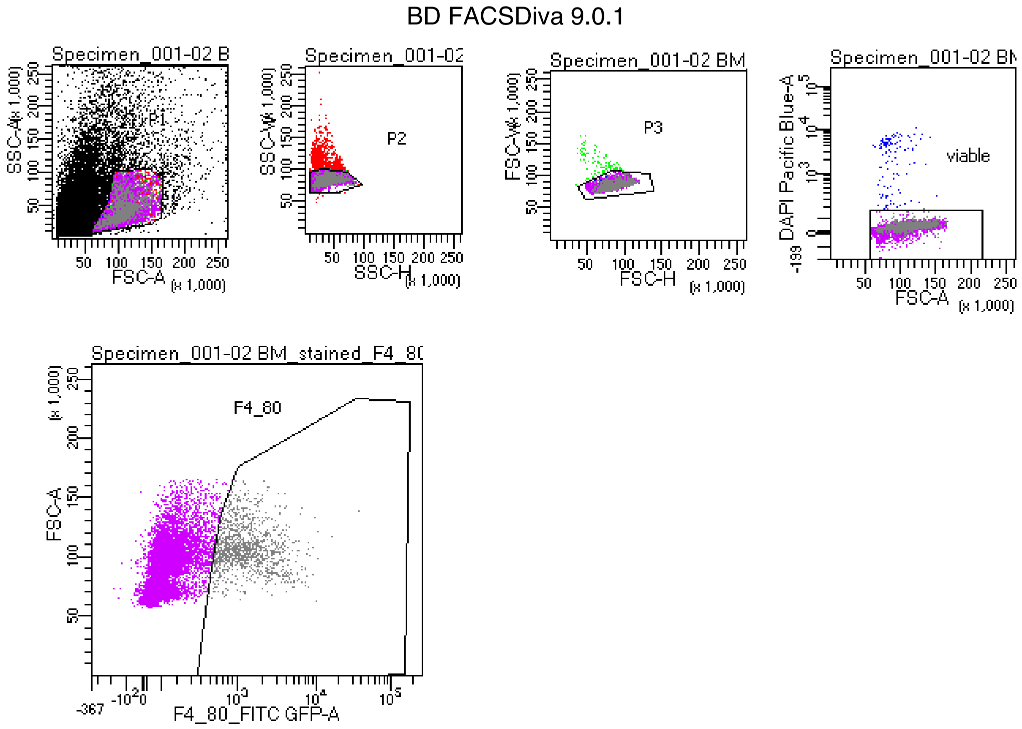

Supplementary Figure 3

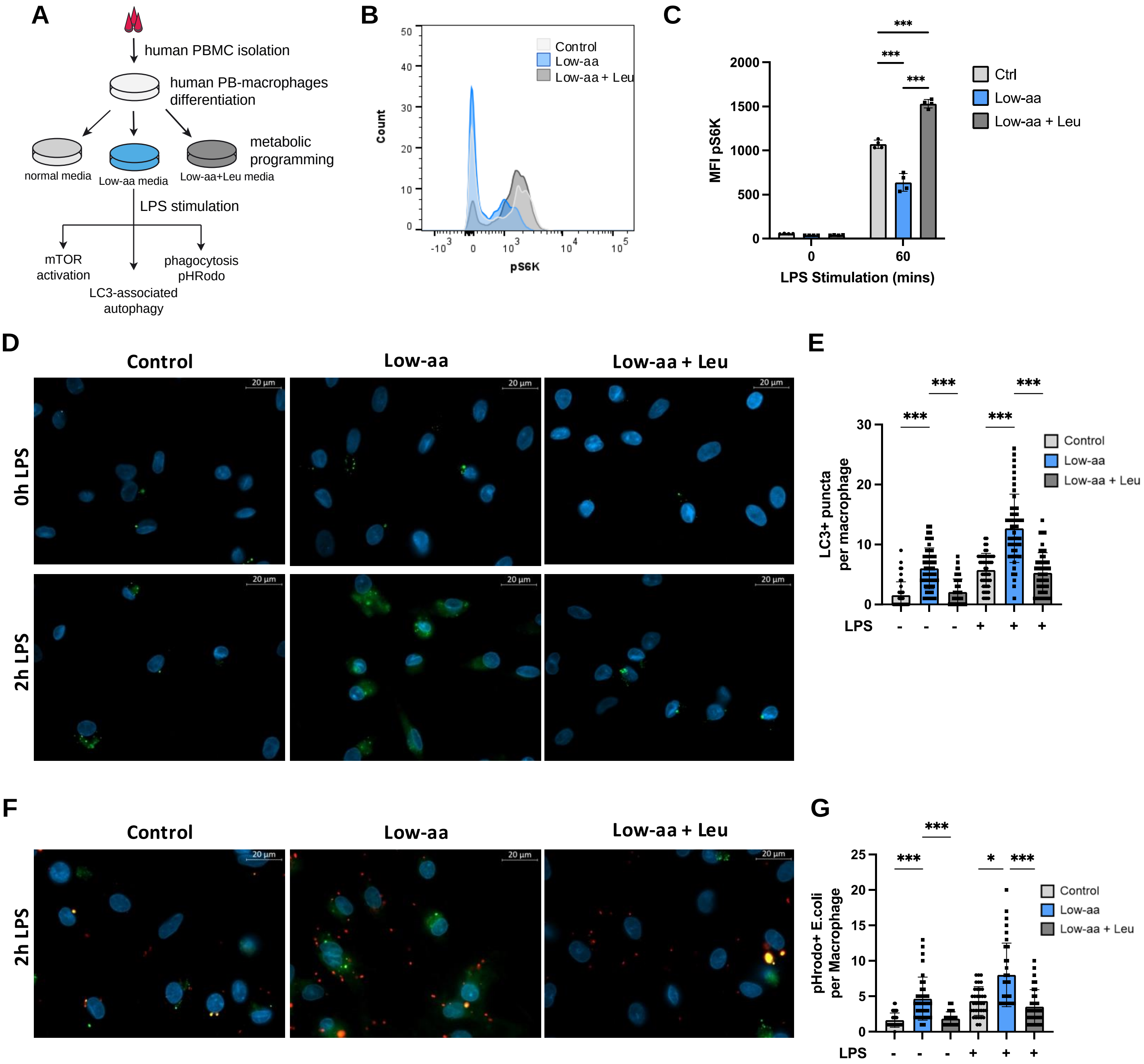

Supplementary Figure 4

Supplementary Figure legends

Supplementary Figure 1. Gating strategy for FACS-sorting of liver immune cells

Cells were gated for size using FSC and SSC, next single cells were chosen using SSCW and SSC-H, to select viable cells only propidium iodide (PI) events were included in the gate. Within this gate only cells positive for CD45 (Pacific Blue+) were sorted.

Supplementary Figure 2. Feature plot depicting marker gene expression in cell clusters.

(A) Basophils: *Cd200r3*, *Cyp11a1*, *Gata2,* CD4+ T cells: *Cd4*, CD8+ T cells: *Cd8a*, B cells: *Ms4a1*, NK cells: *Nkg7, Prf1 and Gzmb* **,** Monocytes: *Cd14 and Cxcl10* **,** Neutrophils: *Chil3, Camp and Ltf*, Dendritic cells: *Cd300e and Ly86*, NK cycling genes included: *Birc5, Pclaf, Cdk1, Ccna2, Kif11 (not shown)*. **(B)** Histogram showing the representation of transcriptionally annotated cell types from immune cells isolated from liver. **(C)** UMAP plot representing the cell type distribution of immune cells in normal (left) or LPD (right) immune cells isolated from liver.

Supplementary Figure 3. Gating strategy for FACS-sorting of primary bone marrow F4-80+ macrophages.

Cells were gated for size using FSC and SSC, next single cells were selected using SSC-W and SSC-H followed by FSC-W and FSC-H. To select viable cells only DAPI negative events were included in the gate. Next, F4-80+ cells were selected using fluorescence minus one (FMO) control.

Supplementary Figure 4. Decreased mTOR activation and increased phagocytic potential in human PBMC-derived macrophages that is reversed with leucine supplementation.

(A) *In vitro* experimental set up for human peripheral blood monocyte cells (PBMC)-derived macrophages. **(B)** Representative histogram depicting intracellular mean fluorescence intensity (iMFI) for pS6 kinase and **(C)** further quantification; Ctrl vs Low-aa, $p<0.001$; Ctrl vs Low-aa+Leu, $p<0.001$

and Low-aa vs Low-aa+Leu $p<0.001$, all after LPS, using Two-Way ANOVA with Tukey's multiple comparisons. **(D)** Immunofluorescence (IF) staining for LC3 puncta and **(E)** further quantification per PBMC-derived macrophage; Ctrl vs Low-aa, $p<0.001$; Low-aa vs Low-aa+Leu, $p<0.001$. After LPS, Ctrl vs Low-aa $p<0.001$; Low-aa vs Low-aa+Leu, $p<0.001$ all using Kruskal-Wallis with Dunns correction. **(F)** IF for LC3-in green and pHrodo E. coli beads in lysosome depicted in red and **(G)** further quantification in PBMC-derived macrophages; Ctrl vs Low-aa, $p<0.001$; Low-aa vs Low-aa+Leu, $p<0.001$. After LPS, Ctrl vs Low-aa $p<0.05$; Low-aa vs Low-aa+Leu, $p<0.001$ all using Kruskal-Wallis with Dunns correction. Representative microscopic images are shown from 63x magnification. Values are mean ± SEM.

Supplementary Data 1

	Control untreated	LPD untreated	Control Salmonella (3d)	LPD Salmonella (3d)
**ALT Fig 1B**	26.22	61.32	456.5	123.4
	27.68	55.52	931.1	240.7
	50.02	101.38	328.1	189.6
	39.94	78.66	1220.2	199.7
	44.64	59.56	556.6	137.1
	40.06	89.82	1285.4	195
		55.94		127.1

	Control untreated	LPD untreated	Control Salmonella (3d)	LPD Salmonella (3d)
	223.82	283.38	802.1	365.5
**AST Fig 1B**	207.4	228.82	2735.8	774.3
	168.58	335.64	435.2	728.2
	167.62	181.84	1840.1	567.2
	108.46	310.76	858.3	503.8
		475.12	2601.4	647.5
		174.54		824.7

	Control	LPD	LEU
**ALT**			
**Fig 5C**	292.8	86.8	145.7
	291.9	63.3	70.5
	347	13.4	222.5
	553.9	134.2	154
	376	182.9	250.5
	383.4	38.5	133.1
	148	132.8	233.5
	173.3	130.5	112.3
		59.3	56.4
		42.8	145.7
			151.7
			203.6
			285.6

	Control	LPD	LEU
**AST**	1388.4	315.8	784.5
**Fig 5C**	1391.2	150.1	1119.1
	916.4	137	783.6
	919.7	650.3	1237.8
	638.6	578.1	1744.1
	1691.5	343	805.6
	292.8	108.9	731
	783.1	497.4	525.1
		308.3	414.3
		275	716
		299.4	891
			1180.5
			617

Fig 2E heatmap

Gene	Normal_Sal/Normal	LPD_Sal/LPD
**Ccl2**	5.45	4.69
**Ccl5**	5.18	2.74
**Cd14**	7.69	6.13
**Cxcl2**	6.91	5.26
**Cxcl9**	9.42	7.48
**Cxcl10**	7.38	3.38
**Ifng**	8.22	7.53
**Il1b**	5.19	3.64
**Il6**	7.54	5.16
**Il12a**	5.26	3.05
**Il12b**	7.97	5.55
**S100a9**	6.98	4.83
**Tlr2**	5.84	3.75
**Tnf**	7.08	5.15
**Trem1**	6.36	4.98

Fig 3C Pathway analysis

KEGG_pathway	Gene_numbe	Fold_enrichm	FDR
antibacterial_humoral_response	15	22.86	1.42E-01
regulation_of_nuclease_activity	21	16.33	3.70E-01
antimicrobial_humoral_response	32	14.29	9.65E-02
translation	213	10.73	7.31E-12
peptide_biosynthetic_process	221	10.34	7.53E-12
amide_biosynthetic_process	275	8.31	2.58E-10
peptide_metabolic_process	276	8.28	2.21E-10

Fig 3D Dotplot

	avg.exp	pct.exp	features.pl	id	avg.exp.scaled
Tnfaip2	22.64	99.00	Tnfaip2	4_LivSalCHOW	0.70710678
Susd6	3.33	78.70	Susd6	4_LivSalCHOW	0.70710678
Oas1a	1.34	52.13	Oas1a	4_LivSalCHOW	0.70710678
S100a8	571.26	99.75	S100a8	4_LivSalCHOW	0.70710678
Mmp8	44.80	95.24	Mmp8	4_LivSalCHOW	-0.7071068
Fos	7.06	80.95	Fos	4_LivSalCHOW	-0.7071068
Lars2	2.99	73.18	Lars2	4_LivSalCHOW	-0.7071068
Nupr1	2.09	48.12	Nupr1	4_LivSalCHOW	0.70710678
Eif1	18.88	100.00	Eif1	4_LivSalCHOW	0.70710678
Tnfaip21	15.72	97.60	Tnfaip2	4_LivSalLPD	-0.7071068
Susd61	2.30	57.60	Susd6	4_LivSalLPD	-0.7071068
Oas1a1	0.53	19.80	Oas1a	4_LivSalLPD	-0.7071068
S100a81	450.25	99.80	S100a8	4_LivSalLPD	-0.7071068
Mmp81	73.92	99.40	Mmp8	4_LivSalLPD	0.70710678
Fos1	11.20	87.80	Fos	4_LivSalLPD	0.70710678
Lars21	5.15	82.80	Lars2	4_LivSalLPD	0.70710678
Nupr11	1.24	32.20	Nupr1	4_LivSalLPD	-0.7071068
Eif11	12.71	98.00	Eif1	4_LivSalLPD	-0.7071068

Fig 4C MFI

	0 min	60min
Control	1254	6281
	1042	8117
	1152	7479
	939	6160
	1119	
LPD	1040	5147
	1202	3214
	819	6080
	826	5213
		2810

Fig 4D qPCR

	NLRP3	IL1b	HIF1a
Control	1.00	1.00	1.79
	0.06	0.86	1.12
	2.73	1.33	0.86
	0.41	0.78	0.74
		0.64	1.00
Control 3h	8.49	216.49	7.03
	7.94	338.88	4.96
	6.53	248.40	3.00
	6.59	272.67	3.66
Low-aa	1.20	0.49	1.81
	0.85	1.11	1.03
	0.40	0.59	0.99
	0.88	0.57	0.79
	1.08	0.56	1.35
Low-aa 3h	4.74	181.06	3.12
	5.72	168.98	3.51
	4.85	179.90	2.84
	5.34	152.80	2.67
	5.22	181.29	3.75

Fig 4F Puncta

Control	Low-aa	Control + LPS	Low-aa + LPS
4	5	3	29
6	16	2	18
10	10	8	10
4	10	3	19
9	13	9	17
6	9	11	12
8	15	12	31
6	12	6	21
6	9	16	15
8	20	9	14
4	12	15	23
1	25	15	20
4	8	16	21
4	21	25	12
1	5	8	17
6	3	6	17
5	3	13	20
2	7	8	33
5	21	18	16
3	12	20	23
10	18	12	11
0	10	11	34
1	2	6	22
4	8	10	10
10	12	20	23
9	13	7	21
5	12	11	18
3	8	13	11
8	14	4	13
9	12	7	26

Fig 5B Macro

LPD	LPD+Leu
13802	19178
15526	18621
16267	

Fig 5F pS6K

	0	30
Ctrl	142	171
	154	175
	167	200
	138	212
Low-aa	135	153
	159	151
	160	143
		143
Low-aa+Leu	188	185
	177	194
	180	199
	173	

g 5H Puncta

Control	Low-aa	Low-aa + Leu	Control LPS	Low-aa LPS	Low-aa + Leu LPS
5	13	6	3	26	14
5	14	6	7	23	10
0	12	5	8	21	17
8	14	11	5	15	17
0	4	5	10	27	12
4	15	4	6	12	5
4	11	5	12	33	18
6	11	4	6	25	6
7	8	10	7	23	8
0	8	10	6	24	5
0	6	3	9	22	8
0	7	2	8	22	18
2	8	0	7	24	8
5	15	2	14	18	3
4	3	9	15	25	16
0	6	3	8	15	3
3	4	2	12	11	4
7	11	1	15	28	17
3	5	4	6	15	3
5	11	8	13	14	9
1	3	12	18	15	5
2	7	1	17	27	14
3	12	2	14	17	4
5	6	3	13	13	5
3	7	10	16	29	7
4	15	8	8	27	13
1	6	9	9	23	7
5	8	8	7	23	10
1	7	4	12	23	8
6	13	5	16	14	15
1	12	2	7	21	8
1	5	6	11	18	9
1	9	9	13	13	16

Fig 5J Phagocytosis

Control LPS	Low-aa LPS	Low-aa + Leu LPS
3	12	5
2	7	2
3	14	3
0	27	10
0	9	11
0	10	2
1	12	18
9	25	19
4	9	5
2	18	8
9	3	19
5	17	20
2	9	16
6	4	3
7	8	7
15	3	5
10	17	8
14	32	5
10	15	12
1	20	1
2	18	2
9	17	22
5	18	13
6	16	3
1	11	2
2	8	3
4	7	6
6	11	5
20	12	4
2	20	7
9	23	9
8	8	11
4	17	8
12	16	9
1	19	3
3	21	3
9	20	4
3	17	4
8	14	8
17	11	5
9	36	17
12	31	9
16	8	3
9	15	3

20	33	2
17	16	1
8	32	6
5	13	5
7	9	17
5	17	5

Supplementary Data 2

Suppl Fig 1B

	%Normal	%LPD
Neutrophils	0.150692677	0.245084464
Monocytes	0.120723777	0.139296594
NK_cycling	0.175289794	0.102741623
NK	0.136273678	0.14040432
CD4	0.111676562	0.13514262
B	0.147017246	0.070063694
CD8	0.070681368	0.06590972
DC_a	0.048911507	0.056770978
DC_b	0.021769861	0.022708391
Basophils	0.014136274	0.017446691
MyB	0.002827255	0.004430906

			used for plotting on the grap	
Cell_type	CHOW-raw_count	LPD-raw_count	%Normal	%LPD
Neutrophils	533	885	0.15069268	0.24508446
NK_cycling	620	371	0.17528979	0.10274162
NK_cycling	482	507	0.13627368	0.14040432
Monocytes	427	503	0.12072378	0.13929659
CD4	395	488	0.11167656	0.13514262
B	520	253	0.14701725	0.07006369
CD8	250	238	0.07068137	0.06590972
DC_a	173	205	0.04891151	0.05677098
DC_b	77	82	0.02176986	0.02270839
Basophils	50	63	0.01413627	0.01744669
MyB	10	16	0.00282725	0.00443091
total	3537	3611		

Suppl Fig 2C pS6K

LPS treatment (time mins)	0	60
Ctrl	59.7	1033.0
	55.1	1034.0
	52.0	1087.0
	53.7	1133.0
Low-aa	38.4	571.0
	39.3	751.0
	38.1	537.0
	36.0	693.0
Low-aa + Leu	40.1	1530.0
	40.0	1469.0
	39.0	1582.0
	36.9	1545.0

Suppl Fig 2E Puncta

Control	Low-aa	Low-aa + Leu	Control LPS	Low-aa LPS	Low-aa + Leu LPS
1	13	4	7	11	9
9	3	2	7	16	7
0	1	0	4	11	1
0	6	0	11	14	1
1	5	0	2	9	1
4	4	0	5	8	1
0	1	0	8	15	2
0	4	0	1	16	7
0	10	3	7	5	2
3	6	5	4	1	3
5	3	4	10	20	4
1	4	1	5	7	9
6	11	1	4	8	6
4	3	0	4	3	4
7	3	0	1	8	2
0	9	0	5	18	14
2	5	0	9	11	10
1	6	5	4	14	3
1	4	3	2	4	12
2	9	2	7	10	12
2	2	1	8	16	3
0	7	0	9	14	8
0	7	0	7	5	4
0	8	0	1	9	6
6	4	0	8	6	6
2	12	0	8	14	4
0	1	0	3	11	5
0	8	1	3	12	7
1	1	4	7	10	5
2	13	2	8	10	7
6	11	4	3	10	3
4	5	6	10	10	7
1	6	3	11	8	1
2	5	1	8	11	1
2	8	2	8	25	12
0	2	1	3	14	3
0	12	6	2	15	6
0	7	2	5	14	2
0	5	2	7	8	4
0	7	5	6	15	1
0	11	8	1	26	1
0	8	5	2	13	7
0	4	4	8	21	5
0	4	2	6	24	8
0	1	7	8	13	6
0	2	0	7	23	5

0	8	1	4	19	6
0	7	1	5	14	6
0	9	1	8	22	9
		4		13	

Suppl Fig 2G Phagocytosis

Control	Low-aa	Low-aa + Leu	Control LPS	Low-aa LPS	Low-aa + Leu LPS
1	7	2	3	4	5
3	4	2	6	13	2
2	2	3	5	4	2
4	3	2	3	4	3
1	2	4	5	5	3
3	1	1	8	4	2
1	3	2	5	5	1
1	4	1	3	4	2
1	2	1	4	10	1
2	2	2	5	4	1
1	4	3	5	5	2
2	3	1	7	9	3
3	13	3	3	4	1
1	4	1	6	5	3
1	3	4	8	4	1
3	4	4	2	4	4
4	5	2	7	8	10
1	6	1	4	7	7
1	2	1	6	8	2
2	8	1	1	4	4
2	5	2	4	7	3
1	4	3	2	8	3
0	3	3	1	7	4
3	6	1	3	5	6
1	2	1	7	12	8
1	1	1	3	4	9
1	2	1	4	14	2
1	5	2	2	20	3
1	11	2	2	12	4
1	12	2	6	7	3
1	7	1	8	10	1
1	5	2	2	16	1
1	4	1	4	15	6
2	5	1	5	12	7
3	10	1	3	17	5
