# Supplementary material for: Low protein diet protects the liver from Salmonella Typhimurium-mediated injury by modulating the mTOR/autophagy axis in macrophages
Source: Commun Biol. 2024 Sep 30;7:1219. doi: 10.1038/s42003-024-06932-w (PMC11444042; doi:10.1038/s42003-024-06932-w)
Supplement: Supplementary file 2 — Reporting Summary [file 42003_2024_6932_MOESM2_ESM.pdf]

nature portfolio

Corresponding author(s): Naiara Beraza
Stuart Rushworth

Last updated by author(s): Sep 9, 2024

Reporting Summary

Nature Portfolio wishes to improve the reproducibility of the work that we publish. This form provides structure for consistency and transparency in reporting. For further information on Nature Portfolio policies, see our Editorial Policies and the Editorial Policy Checklist.

Statistics

For all statistical analyses, confirm that the following items are present in the figure legend, table legend, main text, or Methods section.

n/a	Confirmed	
☐	☒	The exact sample size (*n*) for each experimental group/condition, given as a discrete number and unit of measurement
☐	☒	A statement on whether measurements were taken from distinct samples or whether the same sample was measured repeatedly
☐	☒	The statistical test(s) used AND whether they are one- or two-sided<br>*Only common tests should be described solely by name; describe more complex techniques in the Methods section.*
☒	☐	A description of all covariates tested
☐	☒	A description of any assumptions or corrections, such as tests of normality and adjustment for multiple comparisons
☐	☒	A full description of the statistical parameters including central tendency (e.g. means) or other basic estimates (e.g. regression coefficient) AND variation (e.g. standard deviation) or associated estimates of uncertainty (e.g. confidence intervals)
☐	☒	For null hypothesis testing, the test statistic (e.g. *F*, *t*, *r*) with confidence intervals, effect sizes, degrees of freedom and *P* value noted<br>*Give P values as exact values whenever suitable.*
☒	☐	For Bayesian analysis, information on the choice of priors and Markov chain Monte Carlo settings
☒	☐	For hierarchical and complex designs, identification of the appropriate level for tests and full reporting of outcomes
☒	☐	Estimates of effect sizes (e.g. Cohen's *d*, Pearson's *r*), indicating how they were calculated

Our web collection on statistics for biologists contains articles on many of the points above.

Software and code

Policy information about availability of computer code

Data collection	R version 4.1.3 for Mac
Data analysis	Seurat package for single cell analysis v3.1, tidyverse 2.0.0, ggrepel 0.9.4

For manuscripts utilizing custom algorithms or software that are central to the research but not yet described in published literature, software must be made available to editors and reviewers. We strongly encourage code deposition in a community repository (e.g. GitHub). See the Nature Portfolio guidelines for submitting code & software for further information.

Data

Policy information about availability of data

All manuscripts must include a data availability statement. This statement should provide the following information, where applicable:

- Accession codes, unique identifiers, or web links for publicly available datasets
- A description of any restrictions on data availability
- For clinical datasets or third party data, please ensure that the statement adheres to our policy

Raw sequencing files are available in the directory PRJEB74911

Research involving human participants, their data, or biological material

Policy information about studies with human participants or human data. See also policy information about sex, gender (identity/presentation), and sexual orientation and race, ethnicity and racism.

Reporting on sex and gender	Obtained samples were anonymised on receipt, hence we do not know the gender/age/race/ethnicity of donors.
Reporting on race, ethnicity, or other socially relevant groupings	NA
Population characteristics	NA
Recruitment	National Health Service Blood and Transplant (NHSBT) provides appropriately consented materials to approved recipients for approved non-clinical uses through a managed, governed service we call "Non-clinical Issue" (or NCI). Hence no participants were actively recruited for this study.
Ethics oversight	Faculty of Medicine and Health Sciences Research Ethics Committee (University of East Anglia) approval 2019/20-097 and NHS Blood and Transplant approval R347.

Note that full information on the approval of the study protocol must also be provided in the manuscript.

Field-specific reporting

Please select the one below that is the best fit for your research. If you are not sure, read the appropriate sections before making your selection.

☒ Life sciences ☐ Behavioural & social sciences ☐ Ecological, evolutionary & environmental sciences

For a reference copy of the document with all sections, see nature.com/documents/nr-reporting-summary-flat.pdf

Life sciences study design

All studies must disclose on these points even when the disclosure is negative.

Sample size	In each experiments we included 5-8 animals/group, exact number is provided in the figure legend. Group size was based on our previous studies using Salmonella infection model (Mistry et al., 2021, Issac-Ten et al., 2020).
Data exclusions	We excluded from the analysis animals, which did not present signs on Salmonella infection (CFU colonies from spleen)
Replication	The observation of low protein diet protecting liver function upon Salmonella infection was observed in all performed experiments.
Randomization	Mice were randomized between treatment groups
Blinding	Equal care was taken for all animals, animal care takers were blinded. For in vitro experiments scientists were not blinded.

Reporting for specific materials, systems and methods

We require information from authors about some types of materials, experimental systems and methods used in many studies. Here, indicate whether each material, system or method listed is relevant to your study. If you are not sure if a list item applies to your research, read the appropriate section before selecting a response.

Materials & experimental systems

n/a	Involved in the study	
☒	☐	Antibodies
☒	☐	Eukaryotic cell lines
☒	☐	Palaeontology and archaeology
☐	☒	Animals and other organisms
☒	☐	Clinical data
☒	☐	Dual use research of concern
☒	☐	Plants

Methods

n/a	Involved in the study	
☒	☐	ChIP-seq
☐	☒	Flow cytometry
☒	☐	MRI-based neuroimaging

Animals and other research organisms

Policy information about studies involving animals; ARRIVE guidelines recommended for reporting animal research, and Sex and Gender in Research

Laboratory animals	Mus musculus, C57BL/6, age 8-10 weeks old
Wild animals	NA
Reporting on sex	Experiments performed in male mice
Field-collected samples	NA
Ethics oversight	All experiments were approved by the Animal Welfare and Ethical Review Body (University of East Anglia). All procedures were carried out following the guidelines of the National Academy of Sciences (National Institutes of Health, publication 86-23, revised 1985) and were performed within the provisions of the Animals (Scientific Procedures) Act 1986.

Note that full information on the approval of the study protocol must also be provided in the manuscript.

Plants

Seed stocks	NA
Novel plant genotypes	NA
Authentication	NA

Flow Cytometry

Plots

Confirm that:

☒ The axis labels state the marker and fluorochrome used (e.g. CD4-FITC).

☒ The axis scales are clearly visible. Include numbers along axes only for bottom left plot of group (a 'group' is an analysis of identical markers).

☒ All plots are contour plots with outliers or pseudocolor plots.

☒ A numerical value for number of cells or percentage (with statistics) is provided.

Methodology

Sample preparation	Immune liver cells were isolated as recently reported (10.1084/jem.20082152)
Instrument	BD FACS Aria Fusion, BD Fortessa, 10X Chromium
Software	FlowJo version 10.0
Cell population abundance	Abundance of sorted populations was evaluated using "post-sort" analysis for CD45+ cells followed by manual counting using trypan blue and Burker chamber prior loading onto 10X
Gating strategy	We applied size selection for immune cells isolated from the liver using FSC and SSC. Next viable cells were selected using FMO for negative cell population for propidium iodide (viable cells). These were evaluated for the expression of CD45 marker using the fluorescent minus one (FMO) control to set the gate for the positive gate, this population was FACS purified for 10X analysis.

☒ Tick this box to confirm that a figure exemplifying the gating strategy is provided in the Supplementary Information.
